# Identification of novel functional sequence variants in the gene for peptidase inhibitor 3

**DOI:** 10.1186/1471-2350-7-49

**Published:** 2006-05-23

**Authors:** Mahboob A Chowdhury, Helena Kuivaniemi, Roberto Romero, Samuel Edwin, Tinnakorn Chaiworapongsa, Gerard Tromp

**Affiliations:** 1 Center of Molecular Medicine and Genetics, Wayne State University School of Medicine, Detroit, MI, USA; 2 Perinatology Research Branch, National Institute of Child Health and Human Development, NIH, DHHS, Bethesda, MD, USA; 3 Department of Obstetrics and Gynecology, Wayne State University School of Medicine, Detroit, MI, USA

## Abstract

**Background:**

Peptidase inhibitor 3 (PI3) inhibits neutrophil elastase and proteinase-3, and has a potential role in skin and lung diseases as well as in cancer. Genome-wide expression profiling of chorioamniotic membranes revealed decreased expression of PI3 in women with preterm premature rupture of membranes. To elucidate the molecular mechanisms contributing to the decreased expression in amniotic membranes, the PI3 gene was searched for sequence variations and the functional significance of the identified promoter variants was studied.

**Methods:**

Single nucleotide polymorphisms (SNPs) were identified by direct sequencing of PCR products spanning a region from 1,173 bp upstream to 1,266 bp downstream of the translation start site. Fourteen SNPs were genotyped from 112 and nine SNPs from 24 unrelated individuals. Putative transcription factor binding sites as detected by *in silico *search were verified by electrophoretic mobility shift assay (EMSA) using nuclear extract from Hela and amnion cell nuclear extract. Deviation from Hardy-Weinberg equilibrium (HWE) was tested by χ^2 ^goodness-of-fit test. Haplotypes were estimated using expectation maximization (EM) algorithm.

**Results:**

Twenty-three sequence variations were identified by direct sequencing of polymerase chain reaction (PCR) products covering 2,439 nt of the PI3 gene (-1,173 nt of promoter sequences and all three exons). Analysis of 112 unrelated individuals showed that 20 variants had minor allele frequencies (MAF) ranging from 0.02 to 0.46 representing "true polymorphisms", while three had MAF ≤ 0.01. Eleven variants were in the promoter region; several putative transcription factor binding sites were found at these sites by database searches. Differential binding of transcription factors was demonstrated at two polymorphic sites by electrophoretic mobility shift assays, both in amniotic and HeLa cell nuclear extracts. Differential binding of the transcription factor GATA1 at -689C>G site was confirmed by a supershift.

**Conclusion:**

The promoter sequences of PI3 have a high degree of variability. Functional promoter variants provide a possible mechanism for explaining the differences in PI3 mRNA expression levels in the chorioamniotic membranes, and are also likely to be useful in elucidating the role of PI3 in other diseases.

## Background

PI3 [Gene ID: 5266] is a member of the 'trappin' gene family [1]. The trappin gene family members are defined by an amino-terminal transglutaminase substrate domain consisting of hexapeptide repeats with the consensus sequence of GQDPVK and a carboxy-terminal four-disulphide bond core. PI3, also known as trappin-2, elafin, elastase specific inhibitor and skin-derived antileukoproteinase (SKALP), is a low-molecular weight, 6 kDa serine protease inhibitor [2], that is capable of inhibiting neutrophil elastase (also known as elastase 2; ELA2; [GeneID: 1991]) and proteinase 3 (PRTN3; [GeneID: 5657]; also known as the Wegener autoantigen, P29). PI3 has been mapped to chromosome 20q12-13.1 [3], and this locus contains 14 genes expressing protease inhibitor domains with homology to whey acidic protein (WAP). Human PI3 gene spans about 11,620 bp and consists of three exons [2,4]. The gene has multiple transcription start sites and the mRNA has been reported to have an unusually short 5'-UTR (5'-untranslated region) [5].

Initially, PI3 was identified in human epidermis of psoriatic patients [6], and later in bronchial secretions from patients with bronchial carcinoma [7] and chronic obstructive pulmonary disease [2], as well as in epidermal [8] and breast tumors [9]. In addition to its antipeptidase role, PI3 has antimicrobial activity and is a component of the innate immune system to protect epithelial surfaces from infection [10-13]. Expression of PI3 can be induced by inflammatory mediators such as tumor necrosis factor (TNF) and interleukin 1 beta (IL1B) [14,15].

In our previous report we identified PI3 as a down-regulated gene in the chorioamnionitic membranes of patients with preterm premature rupture of membranes (PPROM) [16]. In this study, we investigated the possible molecular mechanisms that control the expression of PI3 by carrying out a detailed analysis of the PI3 gene sequences.

## Methods

### Genomic DNA isolation

Blood samples were obtained from 112 healthy unrelated African-American individuals after written informed consent. The collection of samples, and their utilization for research purposes, was approved by the Institutional Review Boards of Wayne State University and the National Institute of Child Health and Human Development, NIH. Genomic DNA was extracted from blood samples using QIAGEN^® ^DNA Blood BioRobot^® ^9604 kit (QIAGEN Inc., Valencia, CA.).

### Direct sequencing of PCR products

Genomic DNA was used as a template to generate three overlapping PCR products of 724 bp, 717 bp and 1,328 bp in size extending from 1,173 bp upstream to 1,266 bp downstream of the translation start site of the PI3 gene [GenBank: NT_011362]. Primers are listed in Table 1. All PCRs were carried out in 100-μl volumes containing 1.5 mM of MgCl_2_, 0.2 mM dNTPs, 0.4 μM of each primer, 3 U of Taq DNA polymerase (Roche Molecular Systems, Inc., Branchburg, NJ) and 100 ng of genomic DNA. A 10 minute initial denaturation at 94°C was followed by 40 cycles consisting of 30 s denaturation at 94°C, 30 s annealing at 50°C to 55°C, and 1 minute extension at 72°C. PCR products were analyzed on 2% agarose gels. PCR products were purified by ultrafiltration (Centricon Centrifugal Filter Devices, Millipore, Bedford, MA), and sequenced by cycle sequencing and dye terminator labeling (ABI^® ^BigDye™ Terminator v1.1 Cycle Sequencing kit, Applied Biosystems, Foster City, CA). Sequencing reactions were purified using gel filtration columns (CENTRI-SEP, Princeton Separation, Adelphia, NJ) and run on 310 or 3700 Genetic Analyzer (Applied Biosystems). Sequences were edited using BioEdit [17]. Fourteen SNPs were genotyped from 112 unrelated individuals and nine SNPs from 24 unrelated individuals (Table 2).

**Table 1:** Oligonucleotide primers used in the study.

**Primer Code**	**Sequence^a^**	**Purpose**	**PCR product (bp)**	**Annealing temperature (°C)**
01F_PI3	tgagaagggtgtgtgaaggaa	PCR and sequencing	724	55
01R_PI3	accactcccagcatcaa	PCR and sequencing	724	55
02F_PI3	gagttttttgcaggaccagg	PCR and sequencing	717	52
02R_PI3	gaacagaaagctgaaatctg	PCR and sequencing	717	50
Seq_P13_1328bp_F	caagctggactgcataaaga	PCR	1328	54
Seq_P13_1328bp_R	cagccttcttttgtgtcttc	PCR	1328	53
Seq_P13_Int1_F	tgcataaagattggtatggc	sequencing	-	52
Seq_PI3_Int2_F	tttaaaccttgggtgtggac	sequencing	-	54
Seq_PI3_Int3_F	gaggtgtaccttccctactc	sequencing	-	54
-1077_A_F	ctctccttgtctcAgtgtattagagtc	gel shift assay	-	-
-1077_G_F	ctctccttgtctcGgtgtattagagtc	gel shift assay	-	-
-1067_A_ F	ctcagtgtattagAgtcgtttttctca	gel shift assay	-	-
-1067_G_F	ctcagtgtattaggGtcgtttttctca	gel shift assay	-	-
+1063_A_F	gtgtattagagtcAtttttctcagaca	gel shift assay	-	-
+1063_G_F	gtgtattagagtcGtttttctcagaca	gel shift assay	-	-
-960_T_F	ggaacccccgtttTcccctttcattactt	gel shift assay	-	-
-960_D_F	ggaacccccgtttcccctttcattactt	gel shift assay	-	-
-911_A_F	gttaatagaccagaccaaAtctcacac	gel shift assay	-	-
-911_G_F	gttaatagaccagaccaaGtctcacac	gel shift assay	-	-
-689_C_F	tgtatacatgataCatgttttctacta	gel shift assay	-	-
-689_G_F	tgtatacatgataGatgttttctacta	gel shift assay	-	-
-675_C_F	atgttttctactaCtttctgattattt	gel shift assay	-	-
-675_T_F	atgttttctactaTtttctgattattt	gel shift assay	-	-
-453_T_F	ttgatgctgggagTggtaaaatgataa	gel shift assay	-	-
-453_G_F	ttgatgctgggagGggtaaaatgataa	gel shift assay	-	-
-338_G_F	gaataaccttcgGtgattcctttctcttct	gel shift assay	-	-
-338_A_F	gaataaccttcgAtgattcctttctcttct	gel shift assay	-	-
-258_A_F	taataagtgagccAgcacttctactct	gel shift assay	-	-
-258_G_F	taataagtgagccGgcacttctactct	gel shift assay	-	-

^a^The nucleotide in upper case is the variant nucleotide.

**Table 2:** Minor allele frequencies of the 23 SNPs detected in the PI3 gene.

	**Location**					
					
**GenBank entry position^a^**	**nt^b^**	**region**	**Non-synonymous substitution**	**MAF**	**rs number**	**MAF in dbSNP**
48460A>G	-1077	promoter		0.107		
48470A>G	-1067	promoter		0.107		
48474G>A	-1063	promoter		0.121		
48577T>Del	-960	promoter		0.103		
48626G>A	-911	promoter		0.009		
48669C>G	-868	promoter		0.138	2267863	
48848C>G	-689	promoter		0.107		
48862C>T	-675	promoter		0.107		
49084T>G	-453	promoter		0.005		
49199G>A	-338	promoter		0.107		
49279A>G	-258	promoter		0.005		
49586C>T	+50	exon 1	T17M	0.107	17333103	0.169
49681C>A	+145	IVS 1		0.107	17333180	0.169
49698T>A	+162	IVS 1		0.455	1983649	0.471
49940C>G	+404	IVS 1		0.083^c^		
49944C>T	+408	IVS 1		0.125^c^		
50105C>G	+569	IVS 1		0.146^c^	16989785	0.056
50163A>G	+627	IVS 1		0.146^c^	17424356	0.169
50287T>A	+751	IVS 1		0.063^c^	6032040	0.176
50495A>C	+959	exon 2	T34P	0.125^c^	2664581	0.156
50659C>T	+1123	exon 2		0.020^c^		
50762C>A	+1226	IVS 2		0.125^c^		
50770C>A	+1234	IVS 2		0.125^c^	17424474	0.152

^a^GenBank accession No. AL049767.12.

^b^Location is with respect to translation start site.

^c^Frequency estimated from 24 unrelated individuals.

### *In silico *search for transcription factor binding sites

The sequences in and around the SNP sites in the promoter region were searched for putative transcription factor binding sites using three different computer programs: TESS [18,19], Alibaba 2.1 [20,21], and MatInspector [22,23]. Default parameters were used as search criteria.

### Electrophoretic mobility shift assays (EMSA)

Oligonucleotides (Table 1) and their complementary strands were designed and purchased as gel purified (IDT, Coraville, IA). Complementary oligonucleotides were annealed to each other to generate double-stranded probes. EMSAs were performed using commercially available HeLa cell nuclear extracts (Promega, Madison, WI) and nuclear extracts prepared from primary amnion cell cultures as previously described [24] since we had previously demonstrated that PI3 protein was produced by a variety of chorioamniotic membrane cell types with the highest amount produced by the amniotic epithelial cells [16]. Primary amnion cell cultures were established using amniotic membranes obtained from women not in labor at term who underwent elective cesarean deliveries for obstetrical indications. All other reagents were purchased from a commercial source and used according to the manufacturer's protocol (Promega, Madison, WI). The concentration of poly(dI-dC) (Amersham Biosciences Corp., Piscataway, NJ) in the reaction was optimized to 0.05 μg/μl to minimize non-specific binding. The concentrations of components in 10 μl reaction mixtures were as follows: 1X binding buffer [without poly(dI-dC)], 3.75 μg of HeLa or amnion cell nuclear extract, 0.05 μg/μl poly(dI-dC) and 50 fmole of ^32^P-labeled double-stranded probe (>50,000 cpm). All the components, except ^32^P-probe, were added to the reaction and incubated for 15 min on ice and 10 min at 20°C, followed by the addition of the ^32^P-labelled probe, and incubation for 20 min at 20°C. For competition experiments, a 100-fold molar excess of unlabeled double-stranded oligonucleotides was added to the reaction mixture prior to the addition of the labeled probe. For supershift experiments, polyclonal antibodies against AP1 (Cat. No. sc-253X and sc-44X; Santa Cruz Biotechnology, Santa Cruz, CA), and GATA1 (Active Motif, Carlsbad, CA) were used. For AP1, after 20 min of incubation at 20°C with ^32^P-labelled probe, 400 ng of corresponding antibodies were added to the reaction and incubated for another 15 min at 20°C. For the GATA1 assay, antibodies were added and incubated for 20 min at 20°C before adding the labeled probe [25]. Samples were run on non-denaturing 6% polyacrylamide gels in 0.5X TBE buffer, at 100 V for 80 min. X-ray film (Kodak, Rochester, NY) was exposed to dried gels 2 to 5 h at -80°C depending on signal intensity.

### Nomenclature for sequence variants and genes

The variants and nucleotides are described following the guidelines of the Human Genome Variation Society (HGVS) [26]. SNPs are described using the genomic sequence AL049767.12 as a reference and numbered relative to the translation start site. Official gene symbols provided by Human Genome Organization (HUGO) Nomenclature Committee (HGNC) were used [27].

### Statistical analyses

Tests for deviations from HWE were performed by using the χ^2 ^goodness-of-fit test. Haplotypes were estimated following expectation maximization (EM) algorithm as implemented in the software Arlequin [28].

## Results

### SNP genotyping and haplotype construction

**Figure 1:**
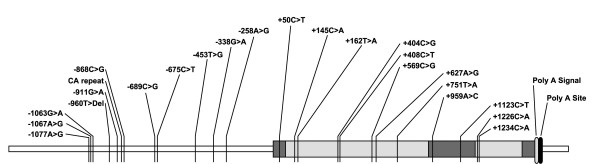
Locations of the 23 SNPs detected in the region from 1,173 bp upstream to 1,266 bp downstream of the translation start site of the PI3 gene. Dark shaded boxes represent the three exons and light shaded boxes represent introns. For more information on the SNPs, see Table 2.

When this study was initiated, only two polymorphisms were known to exist in the PI3 gene, neither of them in the promoter region. We identified 23 SNPs (Table 2 and Fig 1) in the PI3 gene sequences of 24 unrelated individuals by direct sequencing of PCR products that spanned the region from 1,173 bp upstream to 1,266 bp downstream of the translation start site. Of the 23 SNPs, nine are in the dbSNP database [29] (Table 2). Eleven SNPs were located in the promoter region, one in exon 1, seven in intron 1, two in exon 2 and two in intron 2. To obtain more reliable allele frequency estimates, a larger sample of 112 unrelated individuals was genotyped for 14 SNPs. Of the 23 SNPs, three (-911G>A, -453T>G and -258A>G) had minor allele frequencies (MAF) of <0.01 and 20 SNPs had a MAF between 0.02 and 0.46 (Table 2). The genotype frequencies of all SNPs were in HWE. Three SNPs (+50C>T, +959A>C and +1123C>T) altered the codons (Table 2). However, only two SNPs (+50C>T and +959A>C) altered amino acid (Table 2). The T allele at +50 altered the 17^th ^amino acid from threonine to methionine and C allele at +959 altered the 34^th ^amino acid from threonine to proline. The 17^th ^amino acid is part of the signal peptide sequence, whereas the 34^th ^amino acid is part of the proprotein.

Thirteen of the 23 SNPs were in complete linkage disequilibrium (Table 3). Altogether 16 haplotypes were identified (Table 3). PI3_F was the most common haplotype followed by PI3_H and PI3_K.

**Table 3:** Haplotypes constructed with 23 SNPs in the PI3 gene.

Haplotype	Frequency	SD^a^	-1077A>G^b^	-1067A>G^b^	-1063G>A	-960T>Del^b^	-911G>A	-868C>G	-689C>G^b^	-675C>T^b^	-453T>G	-338G>A^b^	-258A>G	+50C>T^b^	+145C>A^b^	+162T>A	+404C>G	+408C>T^b^	+569C>G	+627A>G^b^	+751T>A	+959A>C^b^	+1123C>T	+1226C>A^b^	+1234C>A^b^
PI3_A	0.042	0.0318	A	A	A	T	G	C	C	C	T	G	A	C	C	T	C	C	C	A	A	A	C	C	C
PI3_B	0.021	0.0258	A	A	A	T	G	C	C	C	T	G	A	C	C	T	C	C	C	A	T	A	C	C	C
PI3_C	0.021	0.0154	A	A	A	T	G	C	C	C	T	G	A	C	C	T	G	C	C	A	A	A	C	C	C
PI3_D	0.021	0.0132	A	A	G	T	A	C	C	C	T	G	A	C	C	T	C	C	G	A	T	A	C	C	C
PI3_E	0.021	0.0188	A	A	G	T	G	C	C	C	G	G	A	C	C	A	C	C	C	A	T	A	C	C	C
PI3_F	0.437	0.1006	A	A	G	T	G	C	C	C	T	G	A	C	C	A	C	C	C	A	T	A	C	C	C
PI3_G	0.021	0.0209	A	A	G	T	G	C	C	C	T	G	A	C	C	A	C	C	G	A	T	A	C	C	C
PI3_H	0.125	0.0896	A	A	G	T	G	C	C	C	T	G	A	C	C	T	C	C	C	A	T	A	C	C	C
PI3_I	0.042	0.0355	A	A	G	T	G	C	C	C	T	G	A	C	C	T	G	C	C	A	T	A	C	C	C
PI3_J	0.021	0.0199	A	A	G	T	G	G	C	C	T	G	A	C	C	T	C	C	C	A	T	A	C	C	C
PI3_K	0.083	0.0458	A	A	G	T	G	G	C	C	T	G	A	C	C	T	C	C	G	A	T	A	C	C	C
PI3_L	0.021	0.0156	A	A	G	T	G	G	C	C	T	G	A	C	C	T	C	C	G	G	T	A	C	C	C
PI3_M	0.063	0.0328	G	G	G	Del	G	C	G	T	T	A	A	T	A	T	C	T	C	G	T	C	C	A	A
PI3_N	0.021	0.0177	G	G	G	Del	G	C	G	T	T	A	A	T	A	T	C	T	C	G	T	C	T	A	A
PI3_O	0.021	0.0201	G	G	G	Del	G	C	G	T	T	A	A	T	A	T	G	T	C	G	T	C	C	A	A
PI3_P	0.021	0.0176	G	G	G	Del	G	C	G	T	T	A	G	T	A	T	C	T	C	G	T	C	C	A	A

^a^Standard deviation.

^b^These SNP loci are in complete linkage disequilibrium with each other.

### Effect of SNPs on protein binding

We performed *in silico *searches for putative transcription factor binding sites at the 11 SNP sites in the promoter region of the PI3 gene. Except for one SNP (-675C>T), all other sites showed potential differential binding for at least one transcription factor (Table 4). In other words, the transcription factor was predicted to bind to one of the alleles, but not the other for these 10 SNPs.

**Table 4:** Results from *in silico *searches for putative transcription factor binding sites.

	**Allele**		**Predicted transcription factor(s)^a^**	
		
**Location**	**Major**	**Minor**	**Major allele**	**Minor allele**
-1077	A	G		Adf-2a
-1067	A	G		TBF1
-1063	G	A	GCN4	AP1
-960	T	Deletion	NFATC2	
-911	G	A	SP1, AP1	
-868	C	G		NRC3C1
-689	C	G		GATA1
-675	C	T		
-453	T	G		MAZ
-338	G	A		AP1
-258	A	G	NF1, NFE2, Zta	

^a^The transcription factors, whose binding site is predicted to change by the SNP, are listed here.

**Figure 2:**
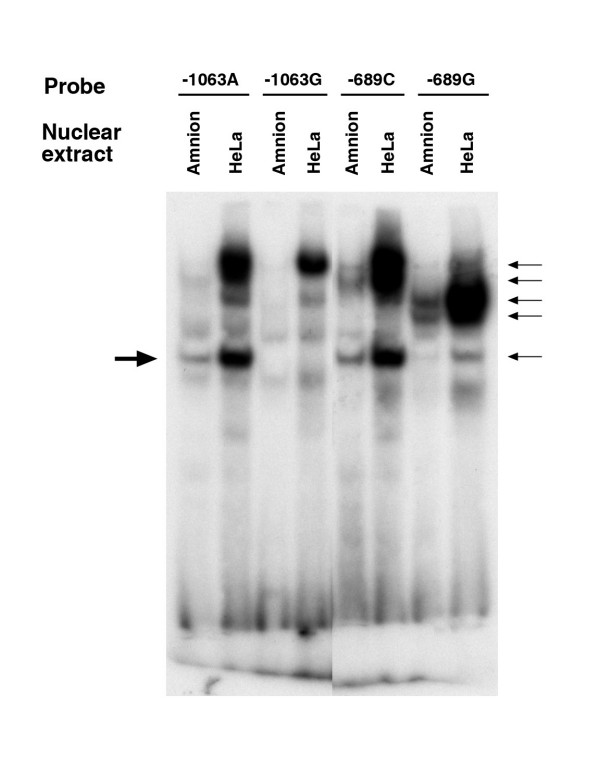
EMSA showing the banding patterns with HeLa and amniotic cell nuclear extracts for -1063A>G and -689C>G sites. The arrows indicate protein-DNA complexes formed when transcription factors bind to their target sites.

**Figure 3:**
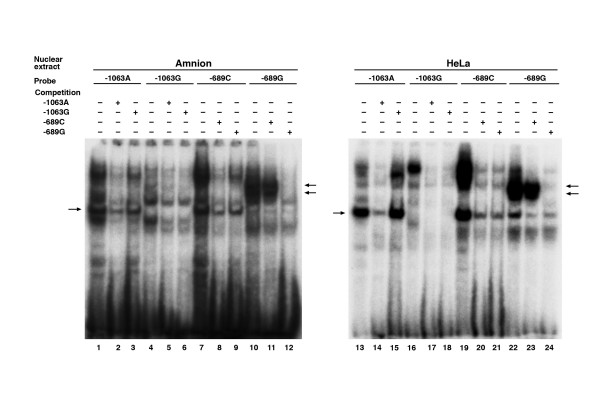
EMSA showing self- and cross-competition for differential binding for -1063A>G and -689C>G sites with amniotic or HeLa cell nuclear extracts. The arrows indicate the differential binding that consistently persisted after cross-competition.

To verify experimentally the differential binding of transcription factors, we conducted electrophoretic mobility shift assays (EMSA) for 10 SNP sites located in the promoter region. Due to the presence of a long stretch of AC-repeats, EMSA was not carried out for the SNP -868C>G. Of the 10 putative sites, six (-1077A>G, -1067A>G, -1063G>A, -960T>Del, -689C>G, -338G>A) showed differential binding by transcription factors in nuclear extracts derived from HeLa cells (not shown), while only two (-1063G>A and -689A>G) showed differential binding using amnion cell nuclear extract (Fig 2). Those SNP sites that did not show differential binding with HeLa cell nuclear extract, also did not show differential binding with amnion cell nuclear extracts. A transcription factor in HeLa cell nuclear extract bound to -960T>Del. There was however, no binding by a transcription factor in amnion cell nuclear extract to this same site. No transcription factor in either HeLa or amnion cell nuclear extract bound to the -258A>G site. Our interest was the transcriptional regulation of the PI3 gene in amnion cells [16]. We, therefore, focused on the two SNPs (-1063G>A and -689C>G, Fig 2) that showed differential binding by transcription factors derived from the amnion cell nuclear extract. For -1063A>G and -689C>G, the banding patterns representing the protein-DNA complexes were similar when using HeLa and amnion cell nuclear extracts, although the band intensities were lower with the latter, probably due to a lower concentration of functional proteins (Fig 2). To determine the specificity of the binding, we used a competition assay (Fig 3). The differential binding that persisted after cross-competition (100-fold) was considered to be due to the SNP. For -1063A>G, one protein-DNA complex persisted after a labeled double-stranded A-probe was competed with double-stranded sequence differing only at the SNP (G instead of A). For -689C>G, two protein-DNA complexes persisted after labeled double-stranded G-probe was competed with a double-stranded sequence differing only at the SNP (C instead of G) (Fig 3).

**Figure 4:**
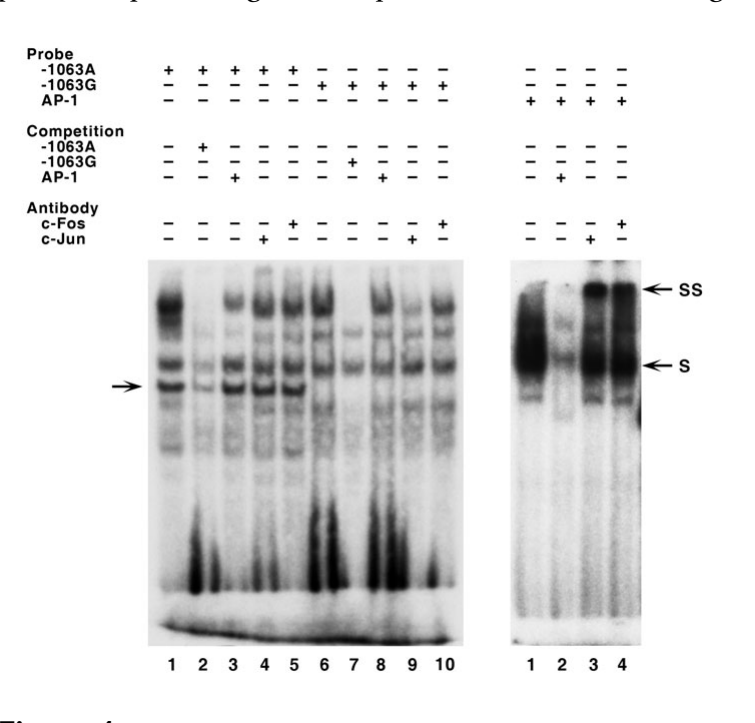
Results of competition and supershift experiments for the -1063A>G site using HeLa cell nuclear extract. S, shift; SS, supershift. Arrow on the left indicates a protein-DNA complex specific to the transcription factor binding to the A-allele.

Our *in silico *search predicted that AP1 was the transcription factor that would bind differentially at the -1063 SNP. To investigate this we used 100-fold excess of a competitor with the consensus sequence for AP1 binding or the anti-AP1 antibody in the reaction. No change in the banding pattern was observed in the competition assay (Fig 4). Similarly, no supershift with anti-c-jun or anti-c-fos antibody was observed for -1063G>A polymorphism using amnion or HeLa cell nuclear extracts (Fig 4). Since a positive control, using consensus AP1 binding sequence, demonstrated a supershift against anti-c-jun and anti-c-fos antibodies (Fig 4), a failure in the supershift was unlikely to be due to technical problems. We, therefore, concluded that the protein that binds to the A probe at nt -1063 does not contain the AP1 epitope.

**Figure 5:**
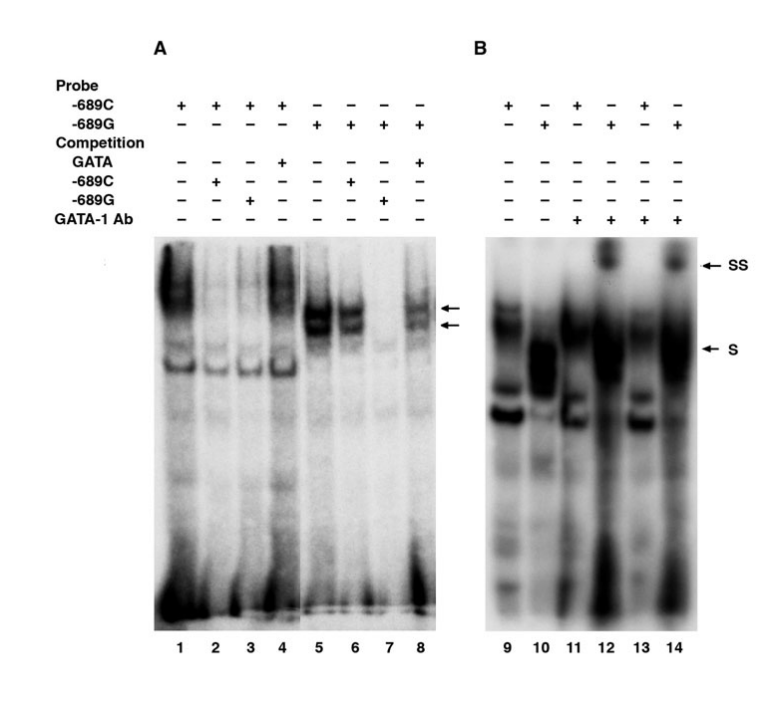
EMSA with amniotic cell nuclear extract for the nt -689C>G SNP site. (A) Competition with -689C, -689G and GATA consensus sequences. The arrows indicate protein-DNA complexes which consistently persisted after cross-competition. (B) Supershift experiment. S, shift; SS, supershift.

For the SNP at nt -689, the transcription factor, whose binding was predicted to change due to the SNP, was GATA1 (Table 5). As shown in Fig 5, a consensus sequence containing the GATA1 binding site was able to compete with the -689G probe (Fig 5A) and a supershift was observed with anti-GATA1 antibody when using amniotic cell nuclear extract (Fig 5B) indicating that GATA1 binds to the G-allele of the -689C>G polymorphism in the promoter region of PI3 gene.

**Table 5:** Previously reported transcription factor binding sites in the PI3 promoter.

**Transcription factor**						**Evidence^c^**		
					
**Gene Symbol**	**Alias**	**Position^a^**	**AL049767.12^b^**	**Author**	**PubMedID**	**IS**	**E**	**DM**
NFKB1	NFκB		48564–48581	King et al. 2003	14521952	+		
				Pol et al. 2003	12542536	+		+
		-479 – -470	49058–49067	King et al. 2003	14521952	+		
		-340 – -331	49197–49206	King et al. 2003	14521952	+		
		-164 – -153	49373–49384	Zhang 1995	7780965	+		+
				Bingle et al. 2001	11472979		+	+
				King et al. 2003	14521952	+		
				Pol et al. 2003	12542536	+		+
JUN	AP1	-545 – -537	48992–49000	Zhang 1995	7780965	+		+
				Zhang 1997	9377579		+	+
				King et al. 2003	14521952	+		
				Pol et al. 2003	12542536	+		+
		-356 – -345	49181–49192	Sallenave et al. 1994	7946401	+		
				Pol et al. 2003	12542536	+		+
SP1		-82 – -74	49455–49463	Zhang 1995	7780965	+		+
				Pol et al. 2003	12542536	+		+
CEBPB	NFIL6	-386 – -378	49151–49159	Pol et al. 2003	12542536	+		+
		-356 – -345	49181–49192	Pol et al. 2003	12542536	+		+
		-307 – -299	49230–49238	Pol et al. 2003	12542536	+		+
		-203 – -194	49334–49343	Sallenave et al. 1994	7946401	+		
				Pol et al. 2003	12542536	+		+
		-126 – -117	49411–49420	Pol et al. 2003	12542536	+		+
OCT1		-590 – -582	48947–48955	Zhang 1995	7780965	+		+
EST1	PEA-3	-484 – -479	49053–49058	Sallenave et al. 1994	7946401	+		

^a^Position is given relative to ATG.

^b^We have mapped the location used in the studies to the current GenBank sequence to standardize the numbering between all studies.

^c^IS, *in silico*; E, EMSA; DM, deletion mapping.

## Discussion

We observed a high degree of polymorphism within the PI3 gene with 23 SNPs detected, 11 of which were located in the promoter region. We found an amino acid substitution, T34P, in the 4^th ^amino acid of the amino terminal-transglutaminase substrate domain, GQDPVK, of PI3. To determine if this SNP has a significant effect on the function of this domain, we searched for the consensus sequence of the transglutaminase substrate in other mammals. A similar sequence domain was identified in seminal vesicle protein I (Semg1) repeats in guinea pig (PROSITE documentation PDOC000282). Semg1 is a clotting protein that serves as the substrate in the formation of the copulatory plug [30]. Covalent clotting of this protein is catalyzed by a transglutaminase and involves the formation of γ-glutamyl-ε-lysine crosslinks. The consensus signature of this consensus sequence was [IVM]-X-G-Q-D-X-V-K-X_5_- [KN]-G-X_3_- [STLV]. The 6^th ^amino acid from the left of this pattern, X, corresponds to the 34^th ^amino acid of the PI3 protein, and appears not to be conserved.

Putative binding of several transcription factors in the promoter region of PI3 gene have been previously reported (Table 5) [5,9,14,15,31]. Except for two, none of these sites were polymorphic in our study. The two consensus sequences of NFKB1 at nt -964 to -956 and nt -340 to -331 identified in previous studies [5,9] contained the -960T>Del and -338G>A SNP sites, respectively, in our study. Based on our *in silico *analysis, the binding of transcription factors NFATC2 and AP1 could be altered by the sequence changes at -960T>Del and -338G>A (Table 4), but with EMSA we did not observe any differences between the two alleles using amnion cell nuclear extract. For -960T>Del we did not observe any binding to a transcription factor using amnion cell extract.

We identified 10 SNPs with alleles that were predicted to have different binding sites for one or more transcription factors by *in silico *searches (Table 4). We tested the predicted differential binding at these sites by EMSA with both HeLa and amnion cell nuclear extracts. HeLa cells are used widely for studying the functionality of promoter polymorphisms. Since PI3 mRNA is down-regulated in chorioamniotic membranes of patients with PPROM [16], we also used a nuclear extract derived from amnion cells. Six of the 10 sites exhibited differential binding to transcription factors with the HeLa extract, in contrast to only two with amnion cell nuclear extract. Since we did not observe supershift at -1063G>A using antibody against AP1 and nuclear extract from either HeLa or amniotic cell line, it is likely that the differential binding was to a transcription factor other than AP1 or the antibody did not have the specific epitope. The presence of AP1 in the nuclear extract of both cell lines was confirmed by the supershift seen when using AP1 consensus probe. We demonstrated the binding of GATA1 to the G allele at the -689C>G site by supershift with an antibody against GATA1.

These findings suggest the involvement of GATA1 in the transcriptional regulation of PI3 gene in amnion cells and provide a possible genetic explanation for the downregulation of PI3 in chorioamniotic membranes from PPROM cases. We have previously demonstrated that the levels of neutrophil elastase (ELA2, [LocusID: 1991]) are increased in the amniotic fluid of patients with PPROM and acute chorioamnionitis [32]. It is therefore plausible to speculate that the production of PI3 in the fetal membranes is to protect the tissue from the damage that could be caused by increased amounts of neutrophil elastase. Our recent study [16] showing decreased expression of PI3 in the chorioamniotic membranes from patients with PPROM supports our hypothesis that patients who are not capable of producing adequate amounts of PI3 may be predisposed to PPROM.

It has been suggested that PI3 is involved in the pathophysiology of many clinical conditions. For example, PI3 was found in the epidermis of patients with psoriasis, but not in normal human epidermis [33]. Higher levels of PI3 were also observed in bronchial secretions from patients with chronic obstructive pulmonary disease [2] and bronchial carcinoma [7], and the expression of PI3 was decreased in breast [9] and in epidermal tumors [8,9]. The SNPs identified here will likely be useful for studying the molecular mechanisms of these diseases.

## Conclusion

A high degree of polymorphism was detected in the PI3 gene with 23 SNPs, 11 of which are in the promoter region. Two SNP sites (-1063G>A and -689C>G) showed differential binding of transcription factors in nuclear extracts derived from both amnion and HeLa cells suggesting possible involvement of these two SNPs in the expression of PI3 gene. As the SNP site at -1063G>A did not bind to the transcription factor AP1 as suggested by *in silico *search, the bound transcription factor may not be in current database and needs to be characterized. Binding of GATA1 to the G allele at the -689C>G site suggests the involvement of GATA1 in the transcriptional regulation of PI3 gene in amnion cells. We have performed a genetic association study with PI3 variants, including the -689C>G variant, and found that it is associated with PPROM [manuscript in preparation]. We also previously demonstrated by immunohistochemistry that many cell types of the chorioamniotic membranes produce PI3 and that PI3 protein is decreased in chorioamniotic membranes from PPROM cases [16]. Together, these lines of evidence provide a plausible genetic explanation for the down regulation of PI3 in chorioamniotic membranes from PPROM cases. Previously the involvement of PI3 in the pathophysiology of many clinical conditions was suggested. The SNPs identified here provide the tools for studying the molecular mechanism of these diseases.

## Competing interests

The author(s) declare that they have no competing interests.

## Authors' contributions

Mahboob A. Chowdhury designed and interpreted experiments; carried out sequencing, EMSAs and *in silico *searches; drafted the manuscript and approved the final version of it.

Helena Kuivaniemi designed and interpreted experiments; carried out PCRs; drafted the manuscript and approved the final version of it.

Roberto Romero provided overall direction to the project; was responsible for the clinical data, revised manuscript and approved the final version of it.

Samuel Edwin established amniotic cell cultures; revised manuscript and approved the final version of it.

Tinnakorn Chaiworapongsa provided clinical data and approved the final version of the manuscript.

Gerard Tromp designed and interpreted experiments; carried out all statistical analyses; provided overall direction to the project; drafted and revised the manuscript as well as approved the final version of it.

## Pre-publication history

The pre-publication history for this paper can be accessed here:


